# Evaluation of a two-image technique consisting of an axial and a coronal image generated by using the rib-flattening application: effect on reading time and diagnostic validity

**DOI:** 10.3906/sag-1908-8

**Published:** 2020-02-13

**Authors:** Koray KILIÇ, Melih AKYÜZ, Emetullah CİNDİL, Nesrin ERDOĞAN, Gonca ERBAŞ, Mehmet ARAÇ

**Affiliations:** 1 Department of Radiology, Faculty of Medicine, Gazi University, Ankara Turkey

**Keywords:** CT, bone reading, rib-flattening, rib metastasis

## Abstract

**Background/aim:**

When reading a chest CT, a radiologist needs to evaluate each rib one by one due to complex curvy shape, which makes reporting a tiresome and time-consuming task. A new curved planar reformat application that flattens ribs on a single plane may find a place in the radiology reporting room. This study aimed to evaluate the effect of a two-image set created by using the rib-flattening application on the performance of a radiologist in detecting sclerotic rib lesions in cancer patients.

**Materials and methods:**

The local Institutional Review Board approved this retrospective study. Two radiologists with different experience levels reviewed chest CT examinations of 106 patients (76 men, 30 women). We divided the patients into group A (n = 54), reviewed by a standard method, and group B (n = 52), reviewed by a standard method and the two-image set created on the rib-flattening application. Reading times, validity indices, and agreement levels with reference data were evaluated for both readers.

**Results:**

The median reading time of the junior examiner significantly decreased with the rib-flattening method (160.5 s vs. 70.0 s; P < 0.001). Diagnostic indices of the senior reader were improved significantly at per patient level (group A, AUC: 0.867; group B, AUC: 0.982; P = 0.046). The new method showed better agreement levels (kappa: 0.69 to 0.96) than the general method (kappa: 0.53 to 0.91).

**Conclusion:**

Based on improved agreement levels, reading times, and diagnostic validity indices we conclude that a two-image set consisting of an axial and a coronal flattened-rib image may be used in conjunction with an ordinary exam.

## 1. Introduction

Ribs are involved in a variety of congenital, metabolic, neoplastic, infectious, and traumatic disorders. Among neoplastic disorders, metastasis is more common than primary bone tumors [1]. Bone metastasis is a frequent complication of cancer. After the liver and lung, bone is the third most common site for cancer spread. Metastasis is most commonly due to breast, prostate, and lung cancer [2]. Chest X-ray is the initial imaging technique when there is a suspicion of bone metastasis. Although it is highly specific, due to lack of sensitivity further evaluation with CT and bone scintigraphy is commonly required [3]. 

When reading a chest CT examination, a radiologist needs to evaluate each rib one by one due to complex curvy shapes and variations. This process makes reporting a tiresome and time-consuming task, causing loss of attention and obviable mistakes [4]. In daily practice, multiplanar image evaluation is the standard of care in rib lesion evaluation. Cho et al. suggested that the evaluation of trauma patients using only axial images resulted in more missed fractures when compared to the use of both axial and coronal reformats in trauma patients [5]. In their study, Alkadhi et al. found that volume rendering as a postprocessing technique is faster than axial images for probing thoracic cage fractures [6]. 

Ringl et al. put forward the use of a new algorithm in the evaluation of trauma patients. They demonstrated that the use of a new algorithm for curved planar images providing flattened-rib images eases the evaluation and increases accuracy [7]. Other authors found that unfolded curved planar images may reduce reading times and increase accuracy in the detection of rib lesions in prostate cancer, breast cancer, and multiple myeloma patients [8–10]. However, there are some drawbacks to the abovementioned technique: the time required to launch the application distracts the attention of the reader, and a necessity for multiple licenses increases the cost for the radiology department. We believe that there is a place for refining the rib-flattening process. We aimed to evaluate the effect of a two-image set created by using the rib-flattening application on the reading times and diagnostic validity of radiologists in detecting sclerotic bone lesions in lung, breast, and prostate cancer patients. 

## 2. Materials and methods

### 2.1. Study design

The local Institutional Review Board approved this retrospective study and waived the need for informed consent. 

We reviewed the hospital records (January 2015 to December 2016) with the following inclusion criteria: (1) adult patients (>18 years); (2) patients with diagnosis of lung, breast, or prostate cancer; (3) patients that had thoracic wall pain with suspected rib metastasis; (4) patients that had chest CT and SPECT or PET CT examinations with an interval of less than a month. A total of consecutive 118 patients were found to meet the inclusion criteria. We divided patients into 2 groups; group A (n = 59) and group B (n = 59). We excluded 12 patients in whom one or more ribs were missing in the two-image set: 5 patients had examinations with movement artifacts, poor reformat quality of 1st rib, or advanced scoliosis (group A, n = 2; group B, n = 3) that precluded readers from reformatting images; 2 patients (group A, n = 1; group B, n = 1) had incompletely scanned lower ribs; and 5 patients (group A, n = 2; group B, n = 3) had previous rib resections. As a result, a total of 106 patients (76 men, 30 women; age range, 23–89 years) constituted the study population. 

**Figure 1:**
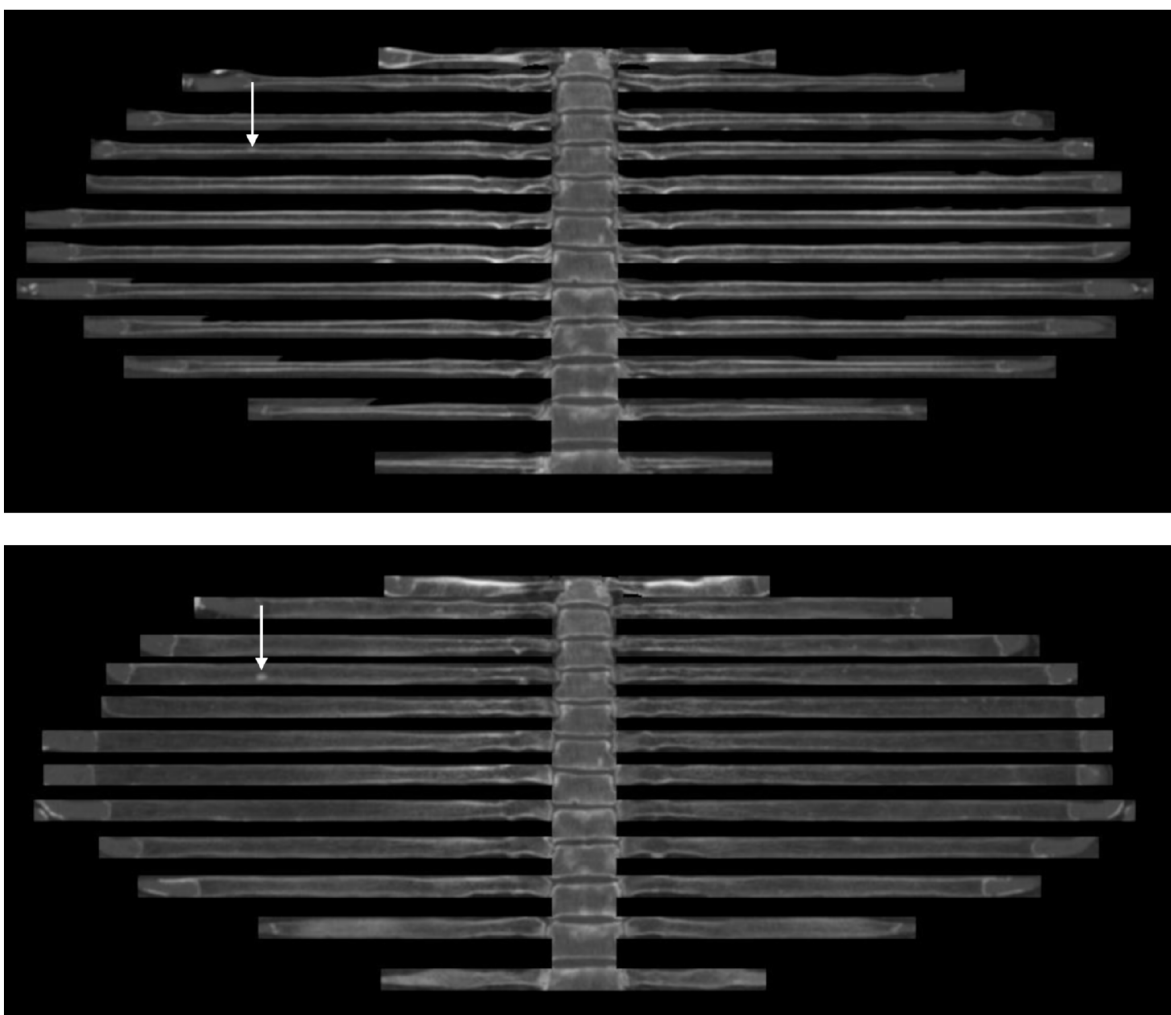
A 66-year-old man who had prostate cancer. Arrows in axial (a) and coronal (b) flattened-rib images show a 5 mm sclerotic foci in the right 4th rib.

**Figure 2:**
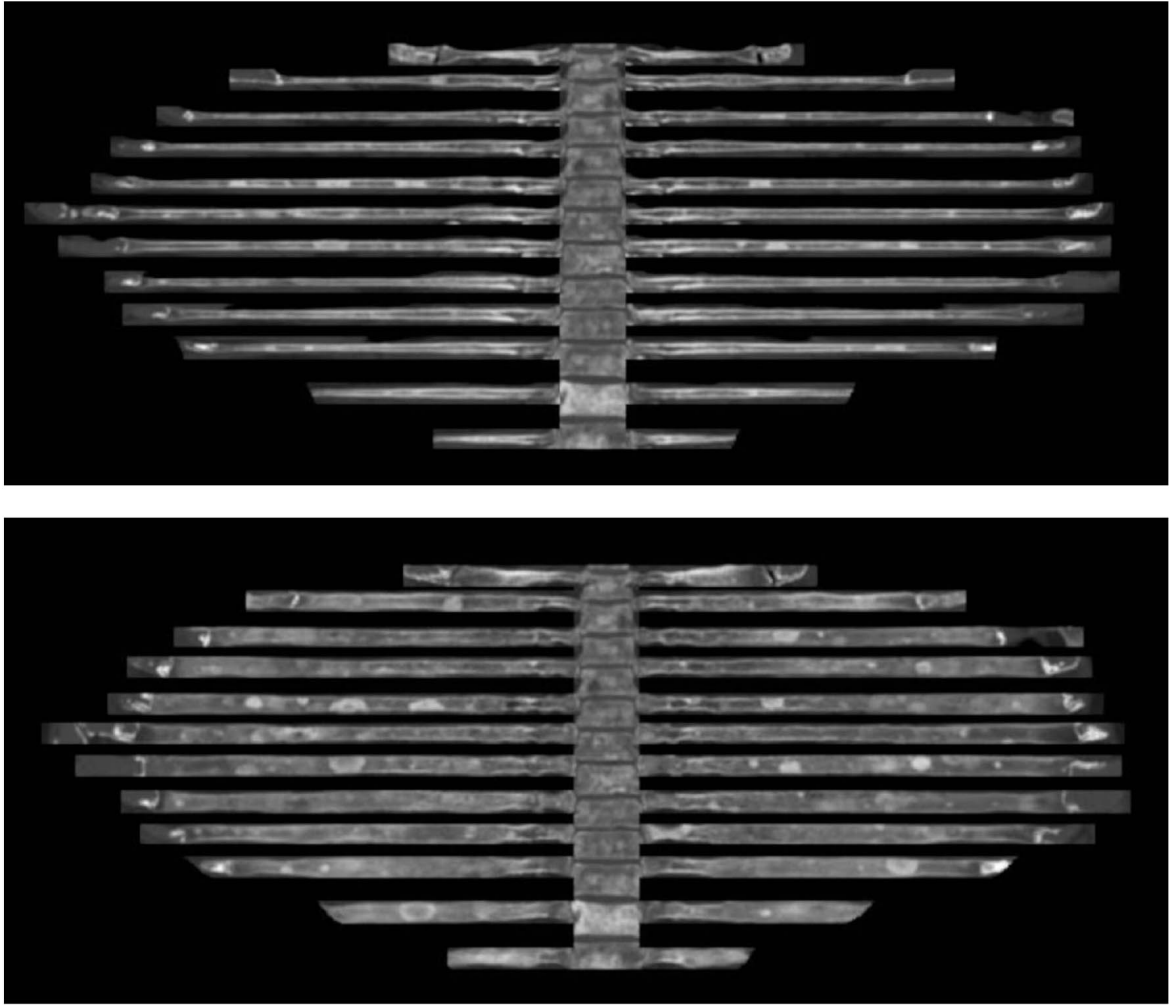
A 74-year-old man had numerous sclerotic bone metastases secondary to prostate cancer on axial (a) and coronal (b) flattened-rib images.

Patients’ CT examinations in group A included standard axial images, whereas the second group (group B) contained a two-image dataset including an axial and a coronal reformatted “flattened-rib” images besides standard images (Figures 1 and 2). We reconstructed these images by using postprocessing—“rib-flattening”—image software (CT Bone Reading, syngo.via, version VB20A, Siemens AG Healthcare, Germany) that automatically flattens the ribs in a single plane and allows a radiologist to evaluate the whole thoracic cage at once. In our department, we use a client-server system that stores applications on a single server. Readers work at client workstations that are connected to the server and use applications with a license.

### 2.2. CT scanning parameters

All examinations were performed by a third-generation 192-section dual-source CT scanner (Somatom Force, Siemens Healthcare, Erlangen, Germany). At the authors’ Department of Radiology, thorax CT protocol parameters were as follows: tube voltage, 120 reference kV; tube current, 210 reference mAs; rotation time, 0.5 s; dose modulation, enabled (Care kV and Care Dose 4D, Siemens Healthcare); volumetric CT dose index (CTDIvol), 5–16 mGy; single collimation width, 0.6 mm; spiral pitch factor, 0.4; slice thickness, 1.0 mm; and convolution kernels, lung (BI57) and mediastinum (Br40d), iterative reconstruction algorithm, ADMIRE strength level 3 out of 5. 

Iohexol (Omnipaque 300 mg/mL, GE Healthcare) was used as a contrast material in all examinations. As a standard procedure contrast material was given at a speed of 4 mL/s by using an IV line (18G) placed in the antecubital vein with a delay of 35 s, followed by 20 mL saline infusion.

### 2.3. Reference preparation and image evaluation

A radiology resident with 3 years of experience and an experienced radiologist with 16 years of experience reviewed the hospital records, anonymized the examinations, and prepared the 2“flattened-rib” images for the group B data set. For comparison, the time that was needed to launch the “rib-flattening” software and that of readily available 2 images was recorded separately in group B. They prepared the reference dataset by doing consensus-reading, using “rib-flattening” software, and interpreting patients’ clinical history with SPECT or PET reports. Two readers, a radiology resident with 4 years of experience and a radiologist with 5 years of experience, who were blinded to diagnosis and did not contribute to the data preparation process, randomly and independently reviewed all examinations in both groups. Readers were asked to spot the ribs with sclerotic lesions. Readers interpreted all the examinations at the same workstation. They were allowed to do multiplane reformatting when they felt necessary for all examinations in both groups A and B. When assessing examinations in group B they also reviewed the two-image data set including flattened-rib images that were prepared by other researchers before reading sessions. Readers did not open the “rib-flattening” software. The number of ribs with lesions and readers’ evaluation times were recorded for all examinations in both groups. 

### 2.4. Statistical analysis

We used SPSS version 17 (SPSS Inc., Chicago, IL, USA) for the statistical analyses. Descriptive data were expressed as mean ± standard deviation, median (25%–75%), n, or percent (%). Normal distribution was checked using the Kolmogorov–Smirnov test and histogram graphs. Categorical variables were compared using the chi-square test or Fischer’s exact test. Between-group comparisons were made using the Mann–Whitney U test or Student’s t-test. Within-group comparisons were made using Wilcoxon signed-rank test or paired t-test. 

A kappa coefficient for agreement was computed for each subgroup with respect to the reference dataset with the following definitions: <0.00, poor; 0.00–0.20, slight; 0.21–0.40, fair; 0.41–0.60, moderate; 0.61–0.80, substantial; and 0.81–1.00, almost perfect [11]. 

We used MedCalc Statistical Software version 18.11.6 (MedCalc Software bvba, Ostend, Belgium; https://www.medcalc.org; 2016) for determining the diagnostic validity of the methods by using indices of sensitivity, specificity, positive predictive value, and negative predictive value. The areas under the receiver operating characteristic (ROC) curves were calculated and compared. A P value of 0.05 was accepted as significant.

## 3. Results

Twenty patients (19%) had breast cancer, 36 patients (34%) had prostate cancer, and 50 patients (47%) had lung cancer. We summarize the clinical and demographical features of the 2 groups in Table 1. No significant difference was observed between the groups in terms of age, sex, cancer type, and scintigraphy findings (P > 0.05 for all). 

**Table 1:** Clinical and demographical features of the patients.

Variables	Group A (n = 54)	Group B (n = 52)	P-value
Age (years)	62.72 ± 12.76	62.50 ± 11.57	0.747
Sex- Male- Female	41 (75.9)13 (24.1)	35 (67.3)17 (32.7)	0.325
Cancer type- Breast- Prostate- Lung	10 (18.5)18 (33.3)26 (48.1)	10 (19.2)18 (34.6)24 (46.2)	0.979
Scintigraphy- Negative- Positive	40 (74.1)14 (25.9)	35 (67.3)17 (32.7)	0.444

*The data are shown as mean ± standard deviation or number of patients (%).

Out of 106 patients, there were 66 patients with sclerotic lesions (62.3%). There was no significant difference in the number of patients with sclerotic lesions between the 2 groups (group A, n = 38 (70.4%) vs. group B, n = 28 (53.8%); P = 0.079). 

Sclerotic lesions were detected in 420 (16.5%) out of 2544 ribs. There was no significant difference in the number of ribs with sclerotic lesions between the 2 groups (group A, n = 205 (15.8%) vs. group B, n = 215 (17.2%); P = 0.338). 

The time that it takes a reader to launch the rib-flattening software was found to be significantly longer than the time to open the axial and coronal flattened-rib images readily available (42.2 ± 2.54 s vs. 6.2 ± 1.17 s; P < 0.001). 

The evaluation time of the junior examiner was significantly longer than that of the senior examiner (P < 0.001 for both groups) (Table 2). The median duration of the junior examiner decreased significantly in group B with the new method (160.5 s vs. 70.0 s; P < 0.001). Although the median duration of the senior examiner decreased, it did not reach significance (66.0 s vs. 58.0 s; P = 0.148). 

**Table 2:** Comparison of the evaluation times of readers according to groups.

Reader	Evaluation times (s)		P¹	Group A	Group B
	Junior	160.5 (93–1123)		70.0 (10–463)	<0.001
Senior	66.0 (32–257)	58.0 (15–443)	P = 0.148
P²	<0.001	<0.017	

The data are shown as median (min–max) in s. ¹Mann–Whitney U test ²Wilcoxon test

The junior reader detected more patients that had sclerotic rib lesions with a lower specificity when the new method was used (Table 3). The overall performance increased, but it was found to be statistically insignificant (group A, AUC: 0.806; group B, AUC: 0.845; P = 0.605). However, we found significant diagnostic improvement in sensitivity and specificity for the senior reader with the new method. The area under the curve was significantly increased (group A, AUC: 0.867; group B, AUC: 0.982; P = 0.046). 

**Table 3:** Diagnostic validity indices: sensitivity, specificity, predictive values, and area under the ROC curve at the per patient level.

	Junior reader		Senior reader		Group A	Group B	Group A	Group B
	Sensitivity	73.7%	85.7%	92.1%	96.4%
Specificity	87.6%	83.3%	81.3%	100.0%
Positive predictive value	93.3%	85.7%	92.1%	100.0%
Negative predictive value	58.3%	83.3%	81.3%	96.0%
AUC*	0.806	0.845	0.867	0.982
Cohen’s kappay	0.534	0.690	0.734	0.961

*Area under the receiver operating characteristic (ROC) curve. yCohen’s kappa coefficient was used to provide a measure of agreement between methods and the reference standard (P < 0.001 for all).

When we considered per rib basis, we found slight improvements in terms of both readers’ sensitivity and senior’s specificity, whereas a minimal decrease in junior’s specificity was encountered (Table 4). Overall performance seems to be increased for both the junior reader (group A, AUC: 0.933; group B, AUC: 0.947; P = 0.371) and the senior reader (group A, AUC: 0.947; group B, AUC: 0.967; P = 0.117) but changes were not statistically significant.

**Table 4:** Diagnostic validity indices: sensitivity, specificity, predictive values, and area under the ROC curve at the per rib level.

	Junior reader		Senior reader		Group A	Group B	Group A	Group B
	Sensitivity	87.3%	90.7%	90.7%	94.4%
Specificity	99.4%	98.7%	98.6%	99.0%
Positive predictive value	96.2%	93.8%	92.5%	95.3%
Negative predictive value	97.7%	98.1%	98.3%	98.8%
AUC*	0.933	0.947	0.947	0.967
Cohen’s kappay	0.901	0.906	0.901	0.938

*Area under the receiver operating characteristic (ROC) curve. yCohen’s kappa coefficient was used to provide a measure of agreement between methods and the reference standard (P < 0.001 for all).

In terms of kappa agreement with the reference method, both readers achieved better results with the new method (group B) compared to the ones with the general method (group A) for both per patient and per rib stratification layers. The new method showed substantial to an almost perfect agreement with kappa ranging from 0.69 to 0.96. The senior reader performed better than the junior reader (Tables 3 and 4). 

## 4. Discussion

In a daily radiology routine, when reading chest CT examinations, axial images are usually found insufficient and multiplanar or 3D volume-rendered images are required [6]. A radiologist needs to track all the ribs through their entire length, especially if the exam belongs to an oncology or trauma patient. It takes a longer time because of their curved shapes. 

In the literature, there are studies describing methods that can flatten anatomic structures like thoracic cage and cranium [7,12]. Seeing the whole structure at once in a single plane helps the reader to identify fractures and lytic or sclerotic lesions faster [9,10,13]. The rib-flattening method also allows the reader to rotate all the ribs through their long axes and generate sagittal, coronal, and axial images through the same point in separate image boxes. Ribs and vertebrae can be numbered automatically [7]. 

However, after implementing this method in our daily practice, we experienced 2 significant issues. One of them was that this commercially available software comes with a limited number of floating licenses, which determines the number of users that can use the software at the same time. If the radiology department has a tight budget, it can become a challenge to afford a large number of licenses, and this software may quickly become a bottleneck in daily practice. We encountered delays due to the limited number of licenses available where a client server system in place. The second issue was the launching and using times of the software. Radiologists find this software useful, but they were reluctant to open it because the process takes a long time and distracts attention from the reading task. 

In this study, we found that the two-image set created using the software that automatically flattens the ribs on a single image plane helped novice readers to evaluate the chest CT examinations faster than the standard method. Our findings are in concordance with the ones that Ha et al. reported in their paper, in which they evaluated the effect of rib-flattening software on a radiologist’s performance [8]. They stated that this specific software helped to improve radiologists’ performance, especially for the inexperienced reader. For the experienced reader, we observed increased diagnostic performance at the per patient layer with a small decrease in reading times, which was statistically insignificant. By using the two-image method, we achieved substantial to almost perfect agreement levels at both layers (per patient/per rib), and for both readers. These agreement levels were better than those of the standard method. However, as opposed to their study, where the readers used all functions of the rib-flattening application, the readers in our study evaluated only the two-image set which consisted of 1coronal and 1 axial flattened-rib image. We found that it was significantly faster to open the two-image set instead of launching the whole application (P < 0.001). 

In daily radiology practice, the two-image dataset included 1 axial and 1 coronal reformatted image to the long axis of ribs, which can easily be prepared by a technician before a reading session. This way, the radiologist does not need to open the application each time and become less distracted. Also, by decreasing the number of users, a radiology department can keep the cost of licenses related to this specific rib-flattening software at a minimum. 

There are limitations to this study. The first limitation is that we excluded lytic lesions. A population of patients with a diagnosis of multiple myeloma may be more suitable for this kind of study. Second, as we included patients with a diagnosis of lung, breast, or prostate cancer who had thoracic wall pain with suspicion of rib metastasis, we came up with a small population. As a result, the prevalence of sclerotic lesions and validity indices was calculated as high. Further studies with a larger population that includes a broader range of diagnoses and clinical status are warranted. 

In conclusion, based on improved agreement levels, reading times, and diagnostic validity indices, we found that a radiologist could benefit from the two-image set when reading a standard CT chest examination.

## Acknowledgments

This research received no specific grant from any funding agency in the public, commercial, or nonprofit sectors. We declare that we have no actual or potential conflicts of interest in relation to this article. 

## Informed consent

The local Institutional Review Board (Institutional Review Board of Gazi University: 2018, Ref. No: 261) approved this retrospective study and waived the need for informed consent.
